# Unilateral hemothorax in a 46 year old South Indian male due to a giant arteriovenous hemodialysis fistula: a case report

**DOI:** 10.1186/1757-1626-1-225

**Published:** 2008-10-07

**Authors:** Shihas Salim, Prasanthi Ganeshram, Amish Dilip Patel, Anita A Kumar, Divya Vemuri, Vijay Jeyachandran, Deepan Rajamanickam, Ghanshyam Palamaner Subash Shantha

**Affiliations:** 1 Department of General Medicine, Sri Ramachandra University, Chennai, India

## Abstract

In a patient undergoing regular hemodialysis through an arteriovenous fistula access, pleural effusion is a known long term complication. However, a unilateral hemothorax is relatively uncommon. Here we report a 46 year old male, end-stage renal disease patient, on maintenance hemodialysis, who presented with a giant brachiocephalic AV fistula in his left arm and progressive breathlessness. Radiological imaging revealed a left sided pleural effusion. Ultrasound guided aspiration revealed a hemorrhagic pleural fluid. A Doppler study of the fistula revealed a high velocity blood flow through the fistula, thereby establishing the cause of the unilateral hemothorax. Ligation of the fistula resulted in complete resolution of the hemothorax. The other possible causes for hemothorax in a dialysis patient are also discussed in this case report.

## Introduction

AV (arteriovenous) fistulas are recognized as the preferred access method for hemodialysis in patients with end stage renal disease. However, they are associated with a separate subset of complications, in addition to those that can potentially occur with hemodialysis. Pleural effusion, with or without hemorrhage, is one among these complications. High flow through the arteriovenous fistula is recognized as an uncommon cause of unilateral hemothorax in such patients. Here we report a patient with a giant AV fistula and a same sided hemorrhagic pleural effusion.

## Case presentation

A 46 year old male, a known hypertensive on treatment for the past 10 years, was diagnosed to have chronic renal failure 3 years ago. Consequently he progressed to ESRD and hemodialysis was initiated in December 2006 for the same, with the creation of a brachiocephalic arteriovenous fistula over the left arm. He has been undergoing regular thrice weekly hemodialysis since then. He is a school teacher by occupation. He had no past history of diabetes mellitus, hypothyroidism, coronary artery disease, or hepatic disease. No past history of Tuberculosis or TB contact. He is married and has two sons. He was a non-smoker and a non-alcoholic.

**Figure 1:**
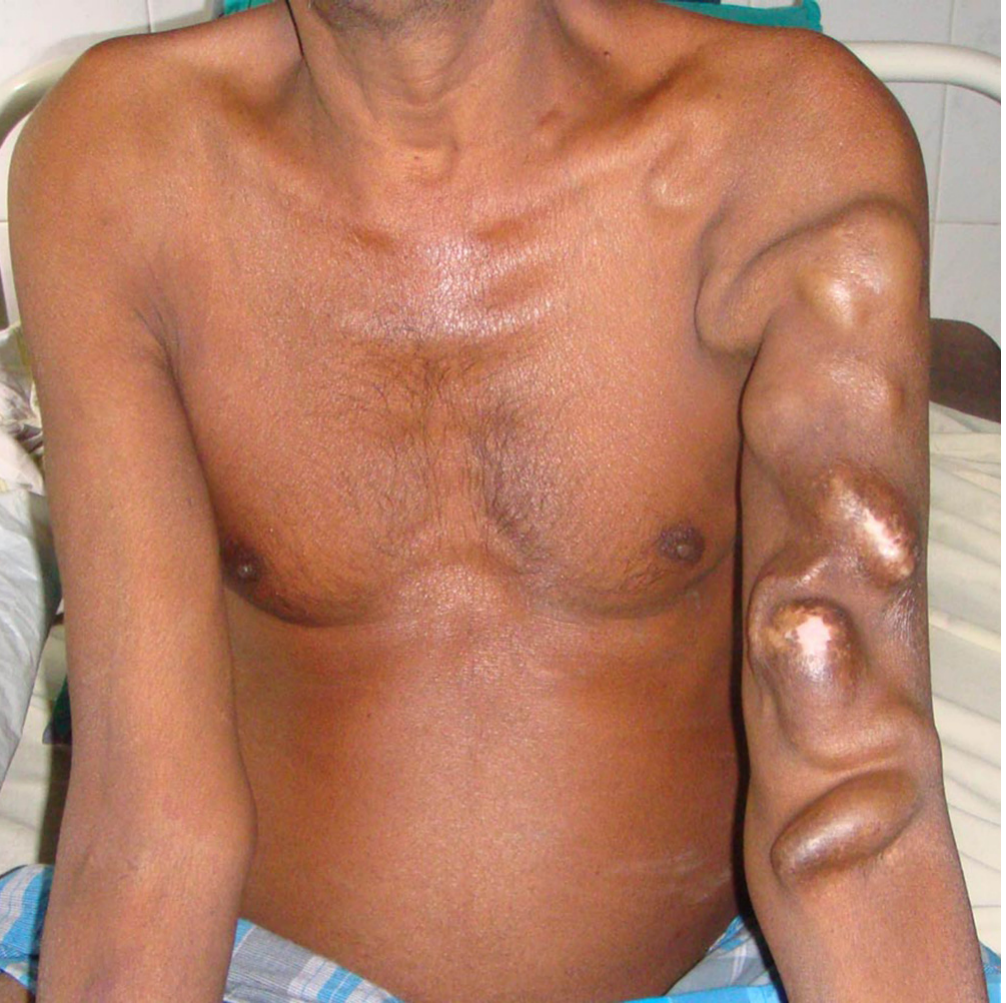
**Giant arteriovenous fistula**. Photograph of the patient's upper body, with the left arm showing a giant arteriovenous fistula extending from the cubital fossa of the left arm up to the left supraclavicular area.

Over the past year, the artificial AV fistula enlarged in size to attain its current dimensions (Figure 1). On 3^rd ^June 2008, he presented to the emergency department of our tertiary care hospital with complaints of progressive breathlessness over the past 15 days. There was no history of associated chest pain, palpitations, syncope, cough, expectoration, or orthopnea.

On examination, he had pallor and bilateral pitting pedal edema. He was afebrile, with a pulse rate of 108/min, BP of 140/80 mmHg, respiratory rate of 28/min. A giant arteriovenous fistula was seen extending from the cubital fossa of the left arm up to the left supraclavicular region (Figure 1). Respiratory system examination revealed stony dullness over the left mammary, infra-axillary and sub-scapular areas on percussion. On auscultation, there was decreased intensity of breath sounds over the above mentioned areas. Normal vesicular breath sounds were heard over all other areas. Other systems examination was unremarkable.

**Figure 2:**
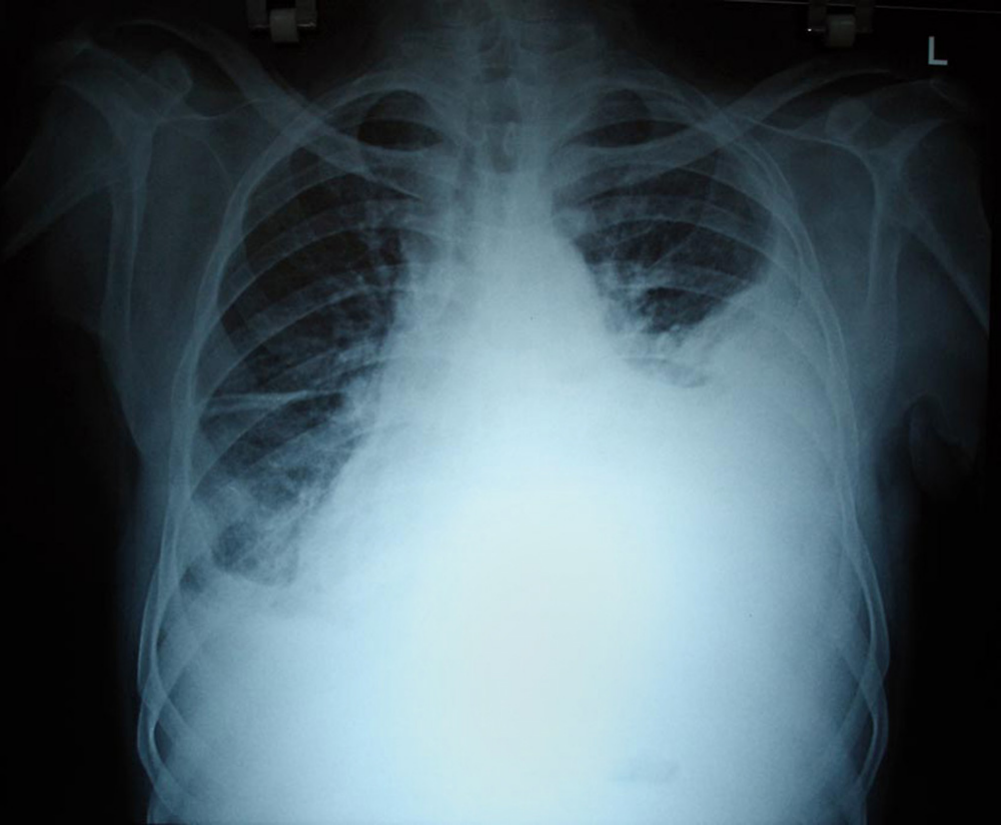
**X-ray chest on admission**. X-ray of the chest (PA-view), showing a left sided massive pleural effusion. This X-ray was taken on admission.

**Figure 3:**
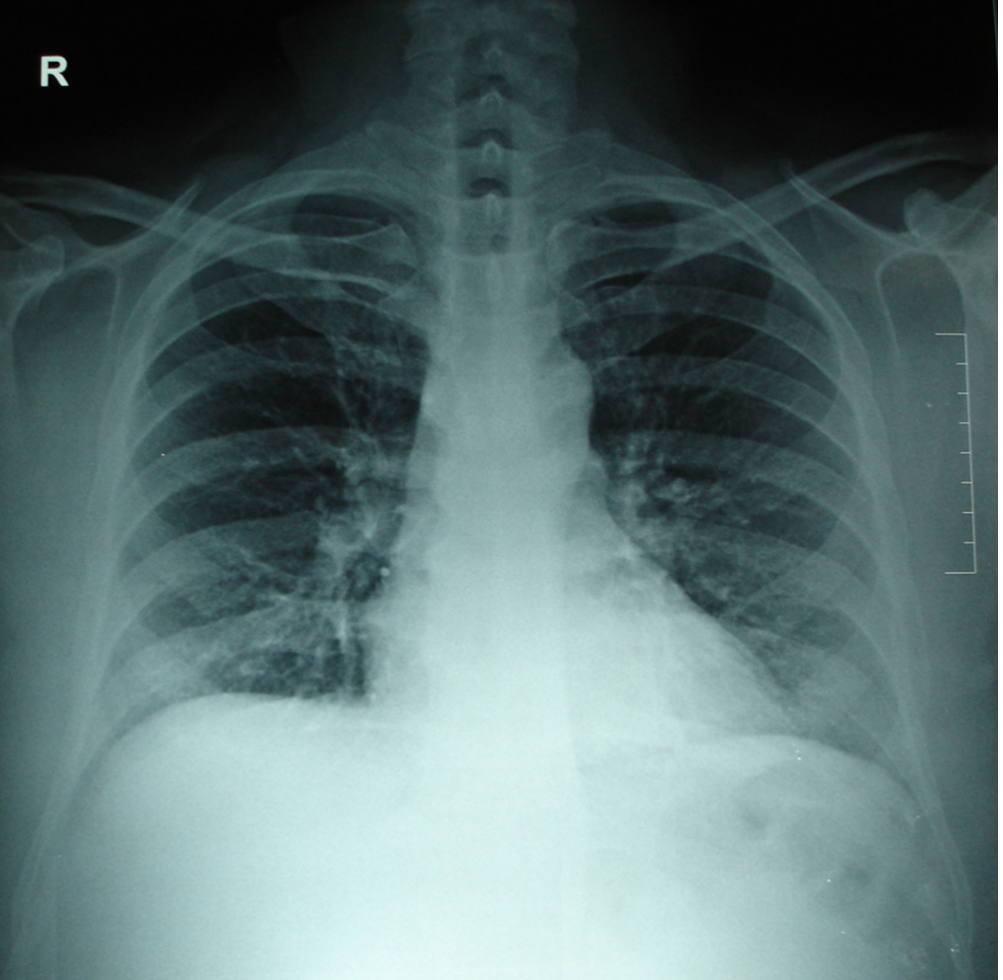
**X-ray chest 6-weeks after ligation of the fistula**. X-ray of the chest (PA-view), taken 6-weeks after ligation of the fistula, showing resolution of the pleural effusion with no recurrence.

His blood investigations showed hemoglobin of 4.5 g/dl, BUN – 13 mg/dl, creatinine- 2.6 mg/dl, with other parameters being within normal limits. His coagulation profile was slightly deranged with a raised INR (Table 1). Chest X-ray revealed a massive pleural effusion on the left side (Figure 2). USG guided pleural aspiration revealed a hemorrhagic fluid. Pleural fluid analysis showed no WBCs, RBC – 5000/mm3 (normocytic 22%, crenated 88%), sugar – 70 mgs/dl, protein – 4.3 g/dl. Doppler study of the AV fistula showed a high peak velocity of 350 cms/sec, with no evidence of thrombosis or stenosis. In order to drain the hemorrhagic pleural effusion, an intercostal drain was placed. Patient was managed with 5 units packed red cell transfusion, oxygen and other supportive measures. With this presentation the possible cause for the left hemothorax was suspected to be due to the AV fistula. Hence after the patient was stabilized, the giant AV fistula was ligated on the 6^th ^day after admission. Following ligation of the fistula, the patient's left sided pleural effusion decreased. The chest drain was removed after 7 days and patient was discharged after 3 more days. On follow up 6 weeks later, the patient did not have a recurrence of pleural effusion (Figure 3). For further hemodialysis, the creation of a new fistula on his right arm has been planned.

**Table 1:** Laboratory investigations

**Parameters**	**Values**
Hb	4.5 g/dl
TC	5060 cells/mm3
Platelet	3.46 lakhs/mm3
INR	1.22
PT	18.3 sec (control-13.9 sec)
PTT	35.0 sec (control-29.6 sec)
Bleeding time	2 min 30 seconds
Clotting time	4 min 25 seconds
D-dimer	<500 ng/ml
BUN	13 mg/dl
Creatinine	2.6 mg/dl
	
**Pleural fluid analysis**:	
WBC	Nil
RBC	5000/mm3 (normocytic 22%, crenated 88%)
Sugar	70 mgs/dl
Protein	4.3 g/dl
PCR for Mycobacterium Tuberculosis	Negative
Cytology for malignant cells	Negative

## Discussion

The incidence of pleural effusion in hospitalized patients receiving long-term hemodialysis is 20.2%; of them 48% have unilateral effusion [1]. Unilateral hemorrhagic pleural effusion can occur secondary to uremia, an emboli arising from the arteriovenous fistula, anticoagulation therapy, subclavian/brachiocephalic vein stenosis/thrombosis, a complication of central venous catheterization, or excessive flow through the AVF access. [2-6]

Uremia causes uremic pleuritis. This evolves into a fibrinous pleuritis which, if untreated, progresses to become a fibrothorax. The pleural effusion takes the form of exudates, which at times is hemorrhagic, and often unilateral. It usually disappears with dialysis and adequate renal replacement therapy, or develops into a fibrothorax. An ultrasonogram may depict septae, fibrinous bands, and debris [2-4]. Considering the clinical presentation of our patient – a giant AV fistula, acute onset massive pleural effusion, ultrasound showing no evidence of septae and adequate control of uremic status, the possibility of uremia being the cause seems unlikely.

In patients on dialysis, hemothorax may occur due to coagulopathy. This can arise secondary to platelet dysfunction of uremia and/or anticoagulant use [2,5,7,8]. Although the coagulation profile in this individual was altered (INR – 1.22), it is more likely that this alteration contributed to the extent of hemorrhage as opposed to it being the primary cause.

Hemothorax could arise as a complication of central venous catheterization. This is usually secondary to malpositioning or malfunctioning of the vascular catheter [2,8]. In our patient, a central venous catheter was not used.

As a result of the increased venous pressure due to the high venous flow through the arteriovenous fistula, stenosis and/or thrombosis of the brachiocephalic and/or subclavian veins can occur. A stenosis of the brachiocephalic vein, in association with high venous flow rates, considerably increase venous pressures in the intercostal and bronchial veins of the left side of the chest. Consequently, local hemodynamics would be altered such that pleural fluid resorption would not occur normally, and excess pleural fluid formation would occur. This leads to unilateral pleural effusion over the same side as the vein stenosis/thrombosis [9]. Doppler study in our patient did not reveal any evidence of stenosis or thrombosis in the brachiocephalic or subclavian vessels.

The other possibility of pulmonary embolism due to a thrombus arising from the AV fistula also seemed unlikely, as there was no evidence of thrombosis in the fistula and the patient's D-dimer levels were within normal limits. Pulmonary embolism usually produces minor pleural effusions unlike the massive effusion in our patient [6,9].

The possibility of the hemothorax occurring secondary to tuberculosis or other infective etiology seems improbable as the patient improved significantly following ligation of the fistula, and had no evidence of recurrence even after 6 weeks. During this entire period he was not given antibiotics or antituberculous therapy. A tuberculous hemothorax resolving without antituberculous therapy is not possible.

A malignant etiology for hemothorax is again unlikely in view of rapid resolution and absence of recurrence of the hemothorax. In addition, the pleural fluid showed no evidence of malignant cells.

There have been reports of hemothorax occurring as a consequence of excessive flow in an AVF access [10]. As a result of the AV fistula, local hemodynamics is altered and the high venous pressure could impede pleural fluid resorption, eventually leading to a same sided pleural effusion with or without hemothorax. This condition usually resolves with ligation of the arteriovenous fistula. [10]. In our patient, this could have been the most likely mechanism, as the patient presented with a high flow giant AV fistula, and once the AV fistula was ligated there was dramatic resolution of the pleural effusion. In addition, on follow-up there was no evidence of recurrence.

## Conclusion

High flow state of an arteriovenous fistula access must be thought of as a differential diagnosis for ipsilateral hemothorax, in a patient on hemodialysis through the same. Dramatic improvement results with ligation of the fistula.

## Patient's perspective

People used to give me odd stares because of the snake-like structure over my arm. When I was admitted, the sight of blood coming out of my chest when the tube was inserted really scared me. I feel a lot more content now that this fistula has been removed. I feel like I have been cured of this problem.

## Abbreviations

AV: arteriovenous; AVF: arteriovenous fistula; ESRD: end stage renal disease; TB: tuberculosis; Hb: hemoglobin; TC: total count; RBC: red blood cells; WBC: white blood cells; BUN: blood urea nitrogen; PT: prothrombin time; PTT: partial thromboplastin time; INR: international normalized ratio; USG: ultrasonogram; PCR: polymerase chain reaction.

## Competing interests

The authors declare that they have no competing interests.

## Authors' contributions

SS, PG, ADP, AAK, DV, VJ, DR, GPSS were involved in the patient care, acquisition of data, analysis and interpretation of data, review of literature, drafting and revising the manuscript.

## Consent

Written informed consent was obtained from the patient for publication of this case report and accompanying images. A copy of the written consent is available for review by the Editor – in – Chief of this journal.

## References

[B1] Bakirci T, Sasak G, Ozturk S, Akcay S, Sezer S, Haberal M (2007). Pleural effusion in long-term hemodialysis patients. Transplantation Proceedings.

[B2] Kim Yookyung, Shim Sung Shine, Shin Jung Hee, Choi Gyu Bock, Lee Kyung Soo, Chin-A Yi, Yu-Whan Oh (2003). Variable pulmonary manifestations in hemodialysis patients. J Korean Radiol Soc.

[B3] Schwartz E (1989). Thoracic manifestations of chronic renal disease. Contemporary Diagn Radiol.

[B4] Galen MA, Steinberg SM, Lowrie EG, Lazarus JM, Hampers CL, Merrill JP (1975). Hemorrhagic pleural effusion in patients undergoing chronic hemodialysis. Ann Intern Med.

[B5] Nasiłowski J, Krenke R, Wewnetrznych Klinika Chorób, Pneumonologii I, Alergologii AM, Warszawie w (2002). Hemothorax with high number of eosinophils following warfarin overdose. Pneumonol Alergol Pol.

[B6] Bensman A, Grimfeld A, Vasmant D, Montagne JP (1982). Massive haemothorax during haemodialysis in a child. Rev Fr Mal Respir.

[B7] Cheah FK, Sheppard MN, Hansell DM (1993). Computed tomography of diffuse pulmonary haemorrhage, with pathological correlation. Clin Radiol.

[B8] Gavelli G, Zompatori M (1997). Thoracic complications in uremic patients and in patients undergoing dialytic treatment: state of the art. Eur J Radiol.

[B9] Wright RobertS, Quinones-Baldrich WilliamJ, Anders AlphaJ, Danovitch GabrielM (1994). Pleural effusion associated with ipsilateral breast and arm edema as a complication of subclavian vein catheterization and arteriovenous fistula formation for hemodialysis. Chest.

[B10] Varan B, Karakayali H, Kutsal A, Ozdemir N (1998). Spontaneous hemothorax in a hemodialysis patient. Pediatric Nephrology.

